# A Case of Wilkie’s Syndrome: Consideration of Alternative Diagnosis in the Setting of Chronic Abdominal Pain

**DOI:** 10.7759/cureus.6074

**Published:** 2019-11-05

**Authors:** John D Adame, Giselle Falconi, Seneca Harberger

**Affiliations:** 1 Family Medicine, Lakeside Medical Center, Belle Glade, USA; 2 Medical Education and Simulation, Aventura Hospital Medical Center, Aventura, USA

**Keywords:** sma syndrome, wilkie's syndrome, close loop referral, rural medicine, rare disease, family medicine, patient safety, graduate medical education, rural health, primary care

## Abstract

Superior mesenteric artery syndrome (SMAS), also known as Wilkie’s syndrome, is an exceedingly rare condition concerning intestinal obstruction. SMAS occurs when the space between the superior mesenteric artery (SMA) and the abdominal aorta narrows, resulting in compression of the duodenum. Functionally, the SMA supplies the distal duodenum, two-thirds of the transverse colon, and the pancreas. The location of the SMA is at about the level of the first lumbar vertebra branching off the anterior portion of the abdominal aorta. Generally, SMAS is due to rapid, excessive weight loss, resulting in the loss of the duodenal fat pad. The loss of the fat pad consequently changes the angle between the abdominal aorta and the SMA, or aortomesenteric angle, causing intestinal obstruction. Typical symptoms of acute cases of SMAS include postprandial abdominal pain, nausea, and vomiting; however, chronic cases may present with vague gastrointestinal symptoms and further weight loss. Herein, we discuss the case of a woman with chronic abdominal pain and previous substantial weight loss in whom we note features consistent with SMAS. Several factors can contribute to the syndrome, but, most commonly, it is observed after sudden, significant weight loss accompanied by nonspecific symptoms such as postprandial epigastric pain, emesis, and anorexia. Given that there is continued debate whether the syndrome even exists, SMAS is usually a diagnosis of exclusion, if diagnosed at all. First-line treatment involves conservative management, but if symptoms become too severe, several proven surgical methods are available. SMAS is a rare condition and is difficult to diagnose, but it should be suspected if clinical manifestations are present. This case illustrates the need for primary care physicians to receive additional training on the recognition of rare diseases to broaden their differentials. Training of this sort is especially crucial for rural family medicine residency programs focused on producing full-spectrum physicians.

## Introduction

We discuss the case of a middle-aged woman with chronic abdominal pain and previous substantial weight loss in whom we note features consistent with superior mesenteric artery syndrome (SMAS). SMAS, also referred to as Wilkie’s syndrome, cast syndrome, aortomesenteric artery compression, etc., is an exceptionally rare condition most commonly affecting younger women [1-2]. SMAS is caused by a compression of the inferior or third part of the duodenum resulting from the narrowing of the space between the superior mesenteric artery (SMA) and the abdominal aorta. Several factors can contribute to the syndrome; most commonly, it is observed after sudden, significant weight loss accompanied by nonspecific symptoms that may include postprandial epigastric pain, emesis, and anorexia. Due to its rarity, SMAS is most often a diagnosis of exclusion, if diagnosed at all, but clinical suspicion should prompt investigation provided awareness of such a syndrome. With a confirmed diagnosis of SMAS, treatment is initially conservative and directed toward symptomatic management and nutritional support. If conservative management fails or if symptoms become too severe, several proven surgical methods are available for a definitive resolution [1-2].

This case highlights that more resources should be allocated to improving and sustaining rural primary care residency programs to produce physicians who will serve rural communities. Despite comprising only 15% of the total outpatient physician workforce, recent data released by the National Rural Health Association estimate that family medicine physicians care for 42% of rural America, which translates to roughly 27 million Americans [3]. Rare diseases often go undiagnosed for many years, and the resulting referral loops can have detrimental effects on patient safety, which will undoubtedly affect quality measures and lead to unnecessary health-care costs [4-5]. 

## Case presentation

An African-American woman in her late forties with a medical history of asthma, type II diabetes, hypertension, diabetic neuropathy, and gastroesophageal reflux disease presented to the Emergency Department (ED) complaining of severe, chronic lower abdominal pain since the past two years. She described the pain as constant cramping and wringing, and localized to the left upper and left lower quadrants, and non-radiating, and rated the pain 8 out of 10 on the visual analog pain scale. Associated aggravating factors included food and non-specific body movements. The patient identified no alleviating factors. She admitted to experiencing shortness of breath due to the severity of the pain, and nausea and vomiting (more than three times before arrival). Vomitus was described as bilious and non-bloody. She denied fever, chills, hematuria, melena, hematochezia, chest pain, drug abuse, and treatment for mental health issues. However, she reported intermittent symptoms of depression and anxiety due to her recurrent abdominal pain, as well as continued use of opioids for pain management. Before admission, she expressed concern regarding potential opioid dependence as she did not want to rely on pain medications.

Her presenting vital signs in the ED included blood pressure of 152/111 mm Hg, heart rate of 95 beats per minute, respiratory rate of 20 breaths per minute, oral temperature of 98.4°F, and oxygen saturation of 100% on room air. She reported that her pain was 10 out of 10. In the ED, treatment included pain management, gastrointestinal prophylaxis, anti-nausea, and anti-spasmodic medications.

Of significance concerning laboratory analysis, she had a blood glucose of 375 mg/dL and an alkaline phosphatase of 156 IU/L. A computed tomography (CT) scan of her abdomen/pelvis on admission revealed constipation and a non-specific, non-obstructive gas pattern.

The patient reported having multiple ED visits per month due to her chronic abdominal pain, with frequent accompanying hospitalizations and no resolution of symptoms. She had previously undergone a laparoscopic cholecystectomy and a hysterectomy secondary to uterine fibroids to resolve the pain. In the past year, she had also been evaluated by a gastroenterologist with negative findings from a workup that included both upper and lower gastrointestinal endoscopies.

**Figure 1:**
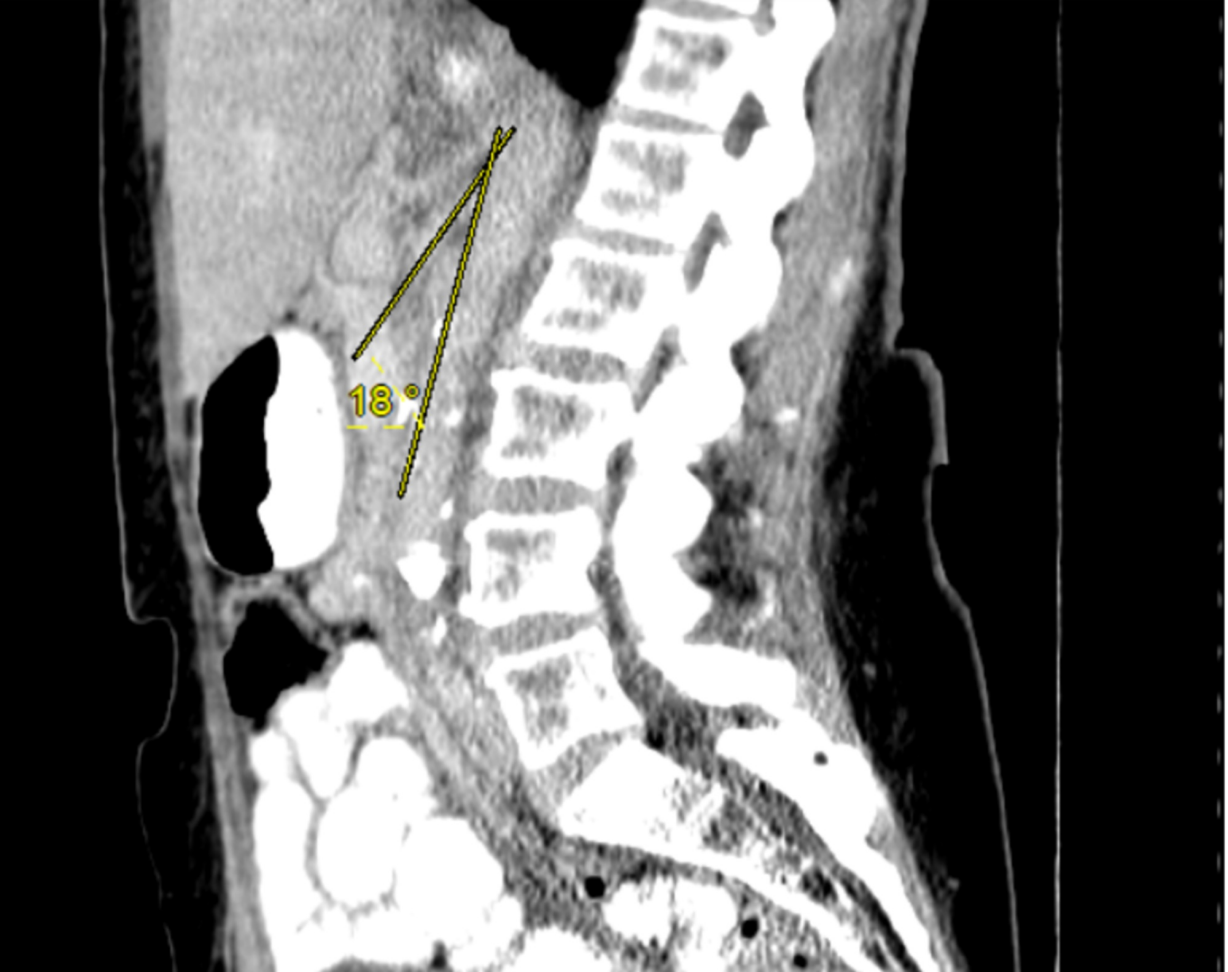
Sagittal CT scan of the abdomen/pelvis with oral and intravenous contrast. The angle between the SMA and the abdominal aorta is normally between 38° and 65°. In SMAS, the angle between the two vessels (i.e., aortomesenteric angle) is less than 25°. CT: computed tomography; SMA: superior mesenteric artery; SMAS: superior mesenteric artery syndrome.

**Figure 2:**
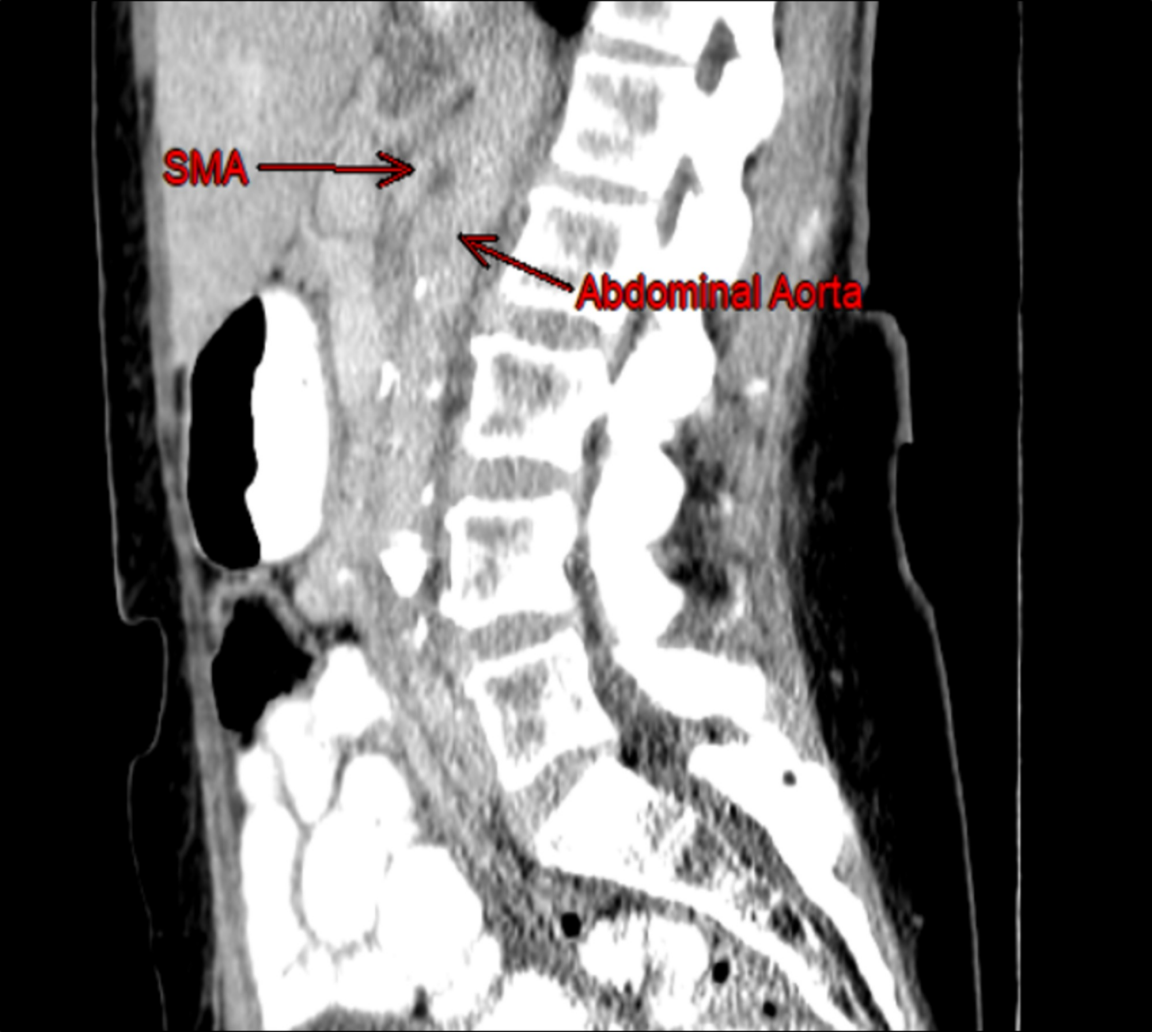
SMA and the abdominal aorta. Sagittal CT scan image is identical to Figure 1. The angle marking has been removed for unobstructed visualization of the vessels. SMA: superior mesenteric artery; CT: computed tomography.

Prior to her diagnosis of diabetes, she weighed approximately 300 pounds. In the two years following the diagnosis of diabetes, she subsequently lost 200 pounds owing to diet and additional lifestyle modifications. The patient’s abdominal pain and ensuing symptoms appeared to coincide with the timing of this substantial, sudden weight loss; given that her symptoms had persisted for over two years, SMAS was considered as a probable cause of her chronic pain. Sagittal CT scan of the abdomen/pelvis, as seen in Figures 1 and 2, revealed what appeared to be a narrowed aortomesenteric angle (AMA) of about 18°, further supporting a diagnosis of SMAS.

CT angiography would have allowed for better visualization in this sagittal view as well as for the aortomesenteric distance to be observed and measured in an axial cut. A surgical consult was ordered, and further diagnostic tests were discussed among the health-care team. Other potential issues such as medication reconciliation and improving care coordination to close referral loops were also considered. Due to our patient’s extensive history, additional differentials included post-cholecystectomy syndrome, gastroparesis, idiopathic intestinal pseudo-obstruction, chronic gastritis, and narcotic bowel syndrome [6].

## Discussion

SMAS is an exceedingly rare diagnosis. On average, studies have shown that a confirmed diagnosis of a rare disease takes approximately five to seven years from the time of symptom onset. SMAS most commonly affects women between 10 and 39 years of age, with a prevalence of about 0.013-0.3% in the general population [1,2,4,5,7]. Due to the presence of a mesenteric fat pad, the angle between the SMA and the aorta (i.e., the AMA) is normally between 38° to 65°, with the duodenum and left renal vein passing through safely protected. The distance between the two is usually 10-28 mm [8]. In SMAS, the loss of the mesenteric fat pad, most commonly seen after sudden, significant weight loss, results in this duodenal compression and subsequent symptoms that may include chronic vomiting, esophageal reflux, and postprandial abdominal pain [9].

A diagnosis of SMAS requires a stepwise approach based on the recognition of presenting symptoms. A barium swallow test can assess for duodenal stricture/dilation. A Doppler ultrasound offers an easy, low-cost, rapid assessment. More sensitive diagnostics include a CT angiography demonstrating an AMA of ≤25°, especially with an aortomesenteric distance of ≤8 mm [2,10,11].

Treatment is generally supportive and limited to symptom management that includes nasogastric tube placement for gastric decompression, patient repositioning to decrease the compression of the duodenum, and pharmacotherapy. Nutritional support is also used to increase adipose tissue to replete the mesenteric fat pad in order to protect the duodenum. If conservative management fails, surgical methods considered include Strong’s procedure (i.e., mobilization of the duodenum by dividing the ligament of Treitz), gastrojejunostomy, or duodenojejunostomy (generally referred to as the superior surgical method) [1,2,9].

## Conclusions

SMAS, as outlined throughout, can pose many challenges and setbacks along the way to a diagnosis and adequate treatment plan. Failure to recognize specific constellations of symptoms, likely due to anchoring bias, scant rare disease awareness, and open referral loops, make the diagnosis of rare diseases an almost insurmountable feat. Therefore, more resources should be allocated to support current rural family medicine residency programs to aid in closing the rural health care gap. As physicians at the front line of medicine, full-spectrum practice along with the knowledge to match is vital for the care of one of the most vulnerable populations in the United States.
